# Increased levels of superoxide dismutase suppress meiotic segregation errors in aging oocytes

**DOI:** 10.1007/s00412-019-00702-y

**Published:** 2019-04-29

**Authors:** Adrienne T. Perkins, Miranda M. Greig, Amrita A. Sontakke, Andrew S. Peloquin, Mark A. McPeek, Sharon E. Bickel

**Affiliations:** 1 Department of Biological Sciences, Dartmouth College, 78 College Street, Hanover, NH 03755 USA; 2 Present Address: Intermountain Healthcare Precision Genomics, 600 S. Medical Center Drive, St. George, UT 84770 USA

**Keywords:** Meiosis, Maternal age effect, Oxidative damage, Reactive oxygen species (ROS), Sister chromatid cohesion, Chromosome segregation

## Abstract

The risk of meiotic segregation errors increases dramatically during a woman’s thirties, a phenomenon known as the maternal age effect. In addition, several lines of evidence indicate that meiotic cohesion deteriorates as oocytes age. One mechanism that may contribute to age-induced loss of cohesion is oxidative damage. In support of this model, we recently reported (Perkins et al. in Proc Natl Acad Sci U S A 113(44):E6823–E6830, 2016) that the knockdown of the reactive oxygen species (ROS)–scavenging enzyme, superoxide dismutase (SOD), during meiotic prophase causes premature loss of arm cohesion and segregation errors in Drosophila oocytes. If age-dependent oxidative damage causes meiotic segregation errors, then the expression of extra SOD1 (cytosolic/nuclear) or SOD2 (mitochondrial) in oocytes may attenuate this effect. To test this hypothesis, we generated flies that contain a UAS-controlled EMPTY, SOD1, or SOD2 cassette and induced expression using a Gal4 driver that turns on during meiotic prophase. We then compared the fidelity of chromosome segregation in aged and non-aged Drosophila oocytes for all three genotypes. As expected, p{EMPTY} oocytes subjected to aging exhibited a significant increase in nondisjunction (NDJ) compared with non-aged oocytes. In contrast, the magnitude of age-dependent NDJ was significantly reduced when expression of extra SOD1 or SOD2 was induced during prophase. Our findings support the hypothesis that a major factor underlying the maternal age effect in humans is age-induced oxidative damage that results in premature loss of meiotic cohesion. Moreover, our work raises the exciting possibility that antioxidant supplementation may provide a preventative strategy to reduce the risk of meiotic segregation errors in older women.

**Electronic supplementary material:**

The online version of this article (10.1007/s00412-019-00702-y) contains supplementary material, which is available to authorized users.

## Introduction

Accurate chromosome segregation during the meiotic divisions is a prerequisite for the formation of a euploid fetus, and chromosome segregation errors in human oocytes are the leading cause of birth defects and pregnancy loss in humans (Nagaoka et al. 2012). During a woman’s thirties, the risk of conceiving an aneuploid fetus increases dramatically, a phenomenon known as the maternal age effect (Hassold and Hunt 2001). Because a woman’s full complement of oocytes is formed before birth, as females age, so do their oocytes. Human oocytes complete meiotic recombination during fetal development and enter an extended diplotene arrest known as dictyate. Spindle assembly and the completion of the first meiotic division are triggered by ovulation, which can occur decades later. Although the molecular mechanisms that contribute to the maternal age effect are likely to be numerous and complex, a unifying model is that age-induced changes that occur in the oocyte during the decades-long dictyate arrest are responsible for the reduced fidelity of meiotic chromosome segregation in older women.

Oxidative damage, caused by reactive oxygen species (ROS), is a hallmark of aging cells, and oxidative stress accompanies many age-related diseases and pathologies (Finkel and Holbrook 2000; Zuo et al. 2015). One such species, the superoxide radical, is produced primarily by mitochondria when an electron leaves the electron transport chain inappropriately (Brand et al. 2004; Murphy 2009). Cells protect themselves from superoxide through the activity of the ROS scavenging enzyme, superoxide dismutase (SOD), and two forms of SOD exist within cells: SOD2 in the mitochondria and SOD1 in the cytoplasm and nucleus (Gertz et al. 2012; Tsang et al. 2014; Wang et al. 2018). Reduced levels of SOD have been correlated with aging (De La Paz and Epstein 1996; Tian et al. 1998; Tsay et al. 2000; Tatone et al. 2006) and the ability of cells to remove oxidatively damaged proteins also declines with age (Hohn et al. 2013). These observations have led to the hypothesis that the accumulation of damaged proteins during the decades-long meiotic arrest of human oocytes contributes to the maternal age effect (Tarin 1995; Tarin 1996; Agarwal et al. 2012; Devine et al. 2012).

Sister chromatid cohesion, mediated by the cohesin complex, is required for accurate chromosome segregation during meiosis (Peters and Nishiyama 2012; McNicoll et al. 2013; Brooker and Berkowitz 2014; Rankin and Dawson 2016). Cohesion not only holds sister chromatids together during meiosis, but also keeps recombinant homologs physically associated until anaphase I (Buonomo et al. 2000; Bickel et al. 2002; Hodges et al. 2005). Cohesive linkages are established during DNA replication (prior to meiotic prophase), and several lines of evidence support the hypothesis that loss of meiotic cohesion during a woman’s lifetime contributes to the maternal age effect (Jessberger 2012; Herbert et al. 2015; MacLennan et al. 2015; Cheng and Liu 2017).

Our recent studies (Perkins et al. 2016) have provided a causative link between oxidative stress and premature loss of meiotic cohesion. When oxidative stress is induced in Drosophila oocytes by the knockdown of either SOD1 or SOD2 during meiotic prophase, arm cohesion is lost prematurely, leading to a significant increase in segregation errors during the first meiotic division. These data are consistent with the hypothesis that oxidative damage incurred during the aging process leads to premature loss of meiotic cohesion and contributes to the maternal age effect in humans.

Given that studying the maternal age effect in humans is experimentally challenging, a model system that recapitulates key aspects of this phenomenon is essential to gain insight into the mechanisms that underlie age-dependent segregation errors. We previously described such an experimental paradigm and demonstrated that when Drosophila oocytes undergo aging, premature loss of meiotic cohesion occurs and results in a significant increase in segregation errors in aged oocytes compared with non-aged control oocytes (Subramanian and Bickel 2008). Notably, these experiments demonstrated that the oocytes most vulnerable to age-induced nondisjunction (NDJ) are those that arrest and age in diplotene, the state in which human oocytes remain arrested for decades (Subramanian and Bickel 2008).

If age-dependent NDJ is due, at least in part, to oxidative damage incurred during the aging process, then the overexpression of SOD1 or SOD2 in aging oocytes may suppress the incidence of chromosome segregation errors. Here, we test this hypothesis by combining our age-dependent NDJ assay with the Gal4/UAS-inducible overexpression of SOD1 or SOD2 during meiotic prophase. We find that the expression of additional SOD1 or SOD2 in Drosophila oocytes that undergo aging significantly reduces the magnitude of age-dependent segregation errors. These data provide compelling support for the hypothesis that the accumulation of oxidative damage during aging contributes to the loss of meiotic cohesion and the maternal age effect in human oocytes. Moreover, our results raise the exciting possibility that nutritional supplementation with antioxidants may provide an effective strategy to counteract age-induced segregation errors in human oocytes.

## Materials and methods

### Creation of UAS-inducible SOD1 and SOD2 transgenes

Full-length SOD1 cDNA (RE52090) and SOD2 cDNA (GH02759) were obtained from the Drosophila Genome Resource Center. The sequences of PCR primers used for amplification and cloning are shown in Table S1. During PCR amplification of both SOD1 and SOD2, an EagI site was introduced near the 5′ end of the open reading frame (ORF), and an XbaI site was introduced near the 3′ end of the ORF to facilitate cloning into the pUASP-attB vector (Takeo et al. 2012). Additionally, a Cavener consensus sequence (CAAA) was introduced directly upstream of the ORF to promote ribosome binding and translation initiation (Cavener 1987). Clones were verified by sequencing and sent to Genetic Services, Inc. (Cambridge, MA) to generate transgenic flies with an insertion on the second chromosome at the *attP40* landing site (Markstein et al. 2008). In addition, the pUASP-attB vector containing no insertion (p{UASP-EMPTY}) was also targeted to the *attP40* site. A series of crosses were performed to generate stocks that contained an *smc1* deletion on the third chromosome (*smc1*^*ex46*^, denoted *smc1Δ* throughout the text) as well as one of the three *p{UASP}* insertions on the second chromosome. Please see Table S2 for the full genotype of these three *p{UASP}; smc1Δ/TM3, Ser* stocks: I-550, I-523, and I-522.

### Age-dependent NDJ assays

**Fig. 1:**
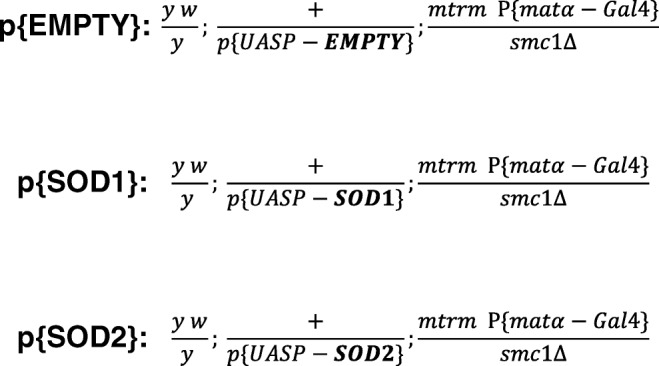
Genotypes used in the age-dependent NDJ assays. Note that for all three genotypes, the indicated p{UASP} insertion resides at the *attP40* landing site on the second chromosome. Flies should differ only in the level of SOD1 or SOD2 expressed in their germlines. Flies are heterozygous for the *smc1* deletion allele (*smc1Δ*) as well as a mutant allele of the *matrimony* (*mtrm*) gene. The expression of the *matα-Gal4* driver begins in region 3 of the germarium, after cohesion has been established, and continues throughout meiotic prophase (Weng et al. 2014)

All flies were reared on standard cornmeal molasses food and kept in a humidified incubator at 25 °C. Table S2 contains a list of fly stocks and genotypes used in this study. To generate the females tested in the age-dependent NDJ assay (see Fig. 1), *y/B*^*S*^*Y; +; D/TM3, Sb* (I-479) males were crossed to *y w; +; mtrm P{matα-GAL4}/TM3, Sb* (W-073) virgins, and *y w/B*^*S*^*Y; +; mtrm P{matα-Gal4}/TM3, Sb* male progeny were crossed to virgins containing a *p{UASP-EMPTY}*, *p{UASP-SOD1}*, or *p{UASP-SOD2}* insertion on the second chromosome and heterozygous for an *smc1* deletion allele on the third chromosome (*p{UASP}; smc1Δ/TM3 Ser*, I-550, I-523, or I-522 respectively). Virgins corresponding to one of the genotypes shown in Fig. 1 (*y w/y; +/p{UASP}; P{matα-Gal4}/smc1Δ*) were collected over an 8–10-h period and kept in vials with yeast overnight. The next day, virgins were split into two groups, transferred to laying bottles and subjected to a four-day regimen to generate either “aged” or “non-aged” oocytes. A detailed description of this experimental regimen is described in Subramanian and Bickel (2008) and the general procedure is outlined in Fig. S1. Briefly, approximately 120 virgins and an equal number of *X^Y, v f B* (C-200) males were placed in a laying bottle to generate females with “non-aged” oocytes. To generate “aged” oocytes, the same number of virgins was added to a laying bottle, but without males. Every 24 h, fresh glucose plates with yeast paste were supplied and the plates that were removed were photographed to document egg laying, or lack thereof. At the end of 4 days, females from each treatment group were placed in vials with fresh *X^Y, v f B* males to assay for meiotic NDJ. A typical experiment yielded 10 vials for each treatment (aged and non-aged) with 10 virgins and 5 fresh males per vial. Parents were transferred to new vials every 24 h to generate “broods” of progeny. Three 24-h broods of progeny were scored for each NDJ test. For each brood, vials were scored and tabulated individually. In addition, aged and non-aged treatments were performed twice for each genotype, and these are included as a block effect in the statistical analysis.

The *X^Y, v f B* males used in the NDJ test allow us to recover progeny that arise from normal female gametes (*X*) as well as those that arise from gametes containing two *X* chromosomes (diplo-*X*), or no *X* chromosomes (nullo-*X*). In addition, we can differentiate these classes of progeny based on their sex and eye phenotype (Bar+ or Bar-). Because all normal progeny can be recovered but only half of the exceptional progeny are viable, %NDJ is calculated using the following formula: [(2 × (Diplos + Nullos))/(*n* + Diplos + Nullos)] × 100 where *n* is the number of total progeny scored.

Statistical analysis was performed using a full three-factor factorial design with blocking. The three main effects included in the statistical model were female genotype (i.e., p{EMPTY}, p{SOD1}, p{SOD2} in Fig. 1), oocyte aging (i.e., aged, non-aged oocytes), and brood number (i.e., first, second, or third brood). All possible interactions between these three main effects were also included in the model. We analyzed three closely related response variables to ensure that the results are robust to the type of response variable used. For each vial, the numbers of normal, diplo-*X*, and nullo-*X* oocytes were counted and %NDJ calculated according to the above formula. The arcsine-square root transform was applied to %NDJ for analysis (Sokal and Rohlf 2012). For each block, we scored ten vials per treatment for the p{EMPTY} and p{SOD1} female treatment combinations and seven vials for the p{SOD2} treatment combinations. In one analysis, we analyzed %NDJ as the response variable in a general linear model using the above statistical model. This analysis was performed using the GLM procedure of SAS version 9.4 (SAS Institute, Inc. 2018). In the second analysis, we used the raw counts of the number of normal oocytes and the number of exceptional oocytes (diplo-*X +* nullo-*X*) arising from nondisjunction as the response variable in a generalized linear model assuming a binomial error and a logit link function. Ideally, one would use a multinomial model for the three oocyte categories (normal, diplo-*X +* nullo-*X*), but given the large number of responses with zeros, we simplified the analysis to a binomial normal vs. exceptional. Finally, we repeated this binomial generalized linear model analysis using the estimated counts used in calculating %NDJ. These two generalized linear models were analyzed using the GLIMMIX procedure of SAS (SAS Institute, Inc. 2018). All three of these analyses gave qualitatively identical inferences, and so we present only the results for %NDJ using the adjusted values and analyzed using the GLIMMIX procedure of SAS. We chose to present the %NDJ calculated from estimated counts because this is a standard method used to describe *X* chromosome NDJ data for Drosophila oocytes and allows direct comparison to our previously published results (Subramanian and Bickel 2008).

### Comparison of *mtrm P{matα-Gal4}* driver chromosomes using an NLS-GFP reporter

Our lab previously generated recombinant chromosomes containing the *mtrm*^*KG*^ allele and the *P{matα-Gal4}* driver on the same chromosome and used a *P{UAS-GFP.nls}* reporter (B-101) to verify the presence of the *matα-Gal4* driver in each recombinant line. Interestingly, different recombinant chromosomes resulted in different levels of Gal4 activity. For most experiments, we use a *mtrm*^*KG*^*P*{*matα-Gal4}* chromosome that results in robust UAS-dependent expression (W-076, (Perkins et al. 2016)). However, to achieve only a modest overexpression of SOD in the current study, we utilized a chromosome for which UAS-inducible expression is much weaker (W-073, or *B*^*S*^*Y* derivative W-107). To present a visual comparison of the relative strengths of the two *mtrm*^*KG*^*P*{*matα-Gal4}* driver chromosomes, we crossed W-076 or W-107 virgins to males that contain the *P{UAS-GFP.nls}* reporter (B-101). Ovaries from *mtrm*^*KG*^*P*{*matα-Gal4}*/*P{UAS-GFP.nls}* females were dissected in 1× PBS and fixed for 5 min at room temperature in 1× PBS containing 4% formaldehyde. Ovarioles were separated using a tungsten needle and mounted on poly-L-lysine-coated coverslips in Slow-Fade Diamond mounting media (ThermoFisher, S36967). We omitted a DNA counterstaining step so that the bleed-through signal of Hoechst was not an issue when viewing a weak GFP nuclear signal. Ovarioles were imaged using a 0.5NA 20X Plan Fluor objective on a Zeiss Axioimager M1 microscope with a Hamamatsu Orca-R2 camera controlled by Volocity 6.3 acquisition software (Quorum Technologies). Image acquisition and processing were identical for the two genotypes.

### Staining and quantification of SOD expression in Drosophila ovarioles

For cytological experiments, the p{EMPTY} and p{SOD1} genotypes shown in Fig. 1 were generated by crossing *P{UASP-EMPTY}; smc1Δ/TM3, Ser (I-550)*, *or p{UASP-SOD1}; smc1Δ/TM3, Ser (I-523*) males to *mtrm P{matα-GAL4}/TM3, Sb (W-107)* virgins. W-107 contains the same *mtrm P{matα-GAL4}* recombinant chromosome as the W-073 stock used for the NDJ experiments. Ovaries were dissected in Grace’s medium (ThermoFisher, 11595030) and fixed for 5 min in a solution containing one part 16% EM grade formaldehyde (Ted Pella, 18505) and two parts Grace’s medium. After rinsing three times in PBS-TX (1× PBS, 0.2% Triton X-100 [ThermoFisher, Surfact-Amps 28,314]) ovaries were permeabilized with two 15-min incubations in 1× PBS containing 0.5% Triton X-100. These and all subsequent incubations were performed with gentle rotation in a deep well dissection dish at room temperature. Following a 1-hour block treatment in 1× PBS-TX containing 0.5% BSA and 5% Normal Donkey serum, ovaries were incubated overnight in PBS-TX containing 0.5% BSA and a 1:500 dilution of Rabbit anti-Sod1 antibody (Abcam, 13498). Ovaries were rinsed three times in 1× PBS-TX and subjected to three 20-min washes in 1× PBS-TX before a 1-hour incubation with Cy3-conjugated Donkey anti-Rabbit antibody (Jackson ImmunoResearch, 711-165-152) diluted 1:400 in 1× PBS-TX containing 0.5% BSA. Following three rinses and three 20-min washes in 1× PBS-TX, ovaries were stained with 1 μg/ml DAPI in 1× PBS for 20 min and washed in 1× PBS containing 0.01% Triton X-100 for 20 min. Ovarioles were separated using a tungsten needle and mounted on poly-L-lysine (Sigma, P8920) coated coverslips in Prolong Diamond (ThermoFisher, P36961).

Single optical sections were collected on a Nikon A1RSi laser scanning confocal using a 1.3NA 40X Plan Fluor objective, 4× frame averaging and sequential scanning (405 and 561 nm lasers). Acquisition settings were identical for the two genotypes. For analysis, images were imported into Volocity 6.5 (Quorum Technologies). To measure the intensity of SOD1 signal in the germline of each egg chamber analyzed, a region of interest was drawn that included the germline area but not the surrounding follicle cells and the average signal intensity was computed in Volocity. 23 p{EMPTY} ovarioles and 36 p{SOD1} ovarioles were imaged, resulting in analysis of 32 egg chambers for p{EMPTY} and 65 for p{SOD1}, ranging from stages 4 through 8. In previous work, we have demonstrated that stages 7–8 are most vulnerable to age-dependent NDJ and these correspond to diplotene (Subramanian and Bickel 2008). The images shown in Fig. S2 have not been contrast enhanced or processed. The graph corresponds to actual intensities in 12-bit images, which have a dynamic range of 0–4095. An unpaired *t* test was utilized to determine statistical significance.

## Results

We have previously described the use of Drosophila oocytes to model the human maternal age effect and demonstrated that meiotic segregation errors increase significantly when Drosophila oocytes undergo aging (Subramanian and Bickel 2008). Although Drosophila oocytes do not normally undergo a protracted prophase arrest, when egg laying is suppressed, the developmental progression of most prophase I stages is halted and oocytes “age” during the arrest (see Fig. S1). Importantly, our prior work found that the oocytes most vulnerable to age-dependent segregation errors were those that arrested and aged in a diplotene-like state (Subramanian and Bickel 2008); this finding is significant because diplotene is the stage at which human oocytes arrest and age for decades. Therefore, our aging regimen and nondisjunction (NDJ) assay provide a useful approach to explore the mechanisms that underlie the maternal age effect in humans.

Our earlier experiments monitored age-induced segregation errors in the genetically sensitized *smc1Δ/mtrm* background (Subramanian and Bickel 2008). These studies demonstrated that aging causes premature loss of cohesion and destabilization of chiasmata which lead to a significant increase in segregation errors in aged versus non-aged oocytes. *smc1Δ* heterozygotes still have sufficient cohesion to ensure accurate segregation during meiosis under normal (non-aged) conditions. However, because the amount of SMC1 protein (and therefore functional cohesin) is reduced approximately two-fold, loss of cohesive linkages during the four-day aging regimen that we utilize is sufficient to result in a significant increase in meiotic segregation errors (Subramanian and Bickel 2008). Heterozygosity for an allele of *matrimony* (*mtrm*) in these experiments is required to disable the achiasmate system which operates in Drosophila oocytes to ensure that bivalents that fail to achieve a crossover still segregate accurately (Harris et al. 2003). However, this back-up system not only prevents the missegregation of achiasmate bivalents, but also those for which the deterioration of cohesion results in chiasma loss. Therefore, a disabled achiasmate system in *mtrm/+* heterozygotes is necessary for us to detect missegregation of bivalents that have lost their chiasmata due to premature loss of arm cohesion. Heterozygosity for a *mtrm* mutation does not disrupt arm cohesion or chiasmata (Xiang et al. 2007).

To test the hypothesis that higher levels of superoxide dismutase may reduce the age-dependent NDJ we observe in *smc1Δ/mtrm* oocytes, we generated and utilized flies containing p{UASP} constructs to express extra SOD1 or SOD2 during meiotic prophase. The three genotypes for which we performed our age-dependent NDJ assay are shown in Fig. 1. To minimize variability due to position effects, each of the constructs was inserted into the *attP40* landing site on the second chromosome (Markstein et al. 2008). p{EMPTY} flies contain an empty vector inserted into this site. Therefore, oocytes of the three genotypes should differ only in the level of SOD1 or SOD2 protein that they express in their germline. For these studies, we utilized a *mtrm P{matα-Gal4}* recombinant chromosome from our stock collection that elicits a much lower level of Gal4-mediated induction than the chromosome we routinely use for UAS-inducible RNAi in the germline (see Fig. S2). Immunoblot analysis of whole ovary extracts did not uncover a reproducible increase in SOD1 or SOD2 protein in p{SOD} ovaries relative to p{EMPTY} ovaries (not shown). However, in immunostaining experiments, we observed a modest (~ 40%) but significant increase in SOD1 protein levels in the germline of p{SOD1} flies compared with that of p{EMPTY} (Fig. S3). Using the same approach with an SOD2 antibody, we were unable to visualize a SOD2 mitochondrial signal; however, because SOD1 and SOD2 constructs were inserted into the same genomic location, we speculate that the overexpression of SOD2 is comparable to that of SOD1. Our NDJ results (below) support the conclusion that SOD2 protein is overexpressed in p{SOD2} oocytes.

**Fig. 2:**
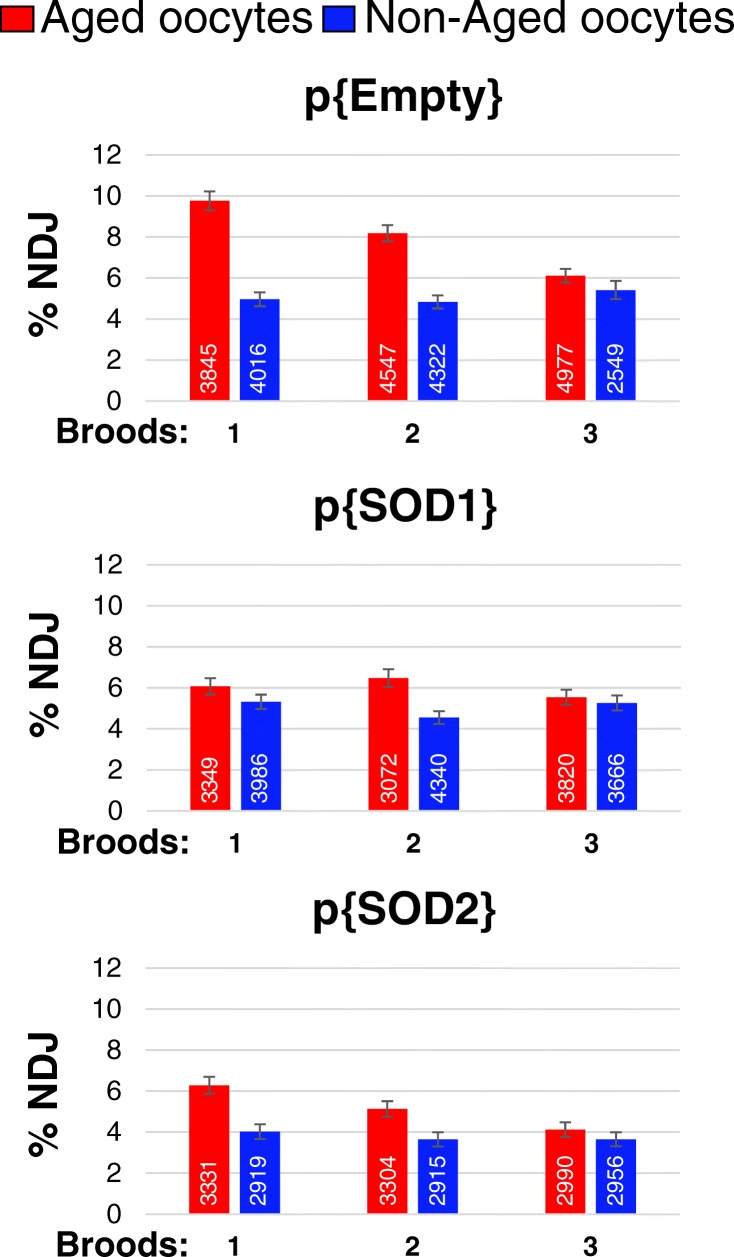
The expression of extra SOD1 or SOD2 during meiotic prophase causes a significant decrease in age-induced segregation errors. %NDJ was measured in the progeny within three 24-h broods. NDJ for oocytes subjected to aging is shown in red; NDJ for non-aged oocytes is shown in blue. The results graphed represent the NDJ values averaged over the two blocks; each block contained one replicate of each of the three genotypes. The total number of progeny scored is shown in white for each bar. Error bars correspond to mean standard error

Figure 2 presents the results of a replicated experiment that explores the effect of genotype on age-dependent NDJ (see Table S3 for the full set of statistical results). For p{EMPTY}, p{SOD1}, and p{SOD2} females, we measured %NDJ in three 24-h broods of progeny arising from aged or non-aged oocytes. Logistical constraints prevented examination of all three genotypes simultaneously. Therefore, we analyzed the data from two different blocks, each of which included one aged and non-aged treatment of each of the three genotypes. Because the results from the two blocks were not significantly different (block *F*_1,305_ = 2.30, *P* = 0.13), we present the %NDJ values averaged over the two blocks.

The data in Fig. 2 indicate that aging causes a significant increase in NDJ (aging *F*_1,305_ = 83.28, *P* < 0.0001). The pattern of NDJ values for aged and non-aged p{EMPTY} oocytes is quite similar to the results we have reported previously for *smc1Δ/mtrm* females (Subramanian and Bickel 2008). Also consistent with our previous data, the level of age-induced NDJ differed across the broods (aging*brood: *F*_2,305_ = 9.22, *P* = 0.0001). For p{EMPTY} oocytes, the age effect was greatest in the first 24-h brood, somewhat decreased in the second 24-h brood, and negligible in the third 24-h brood while the NDJ values for non-aged oocytes were similar in all three broods. We have reported this same outcome for *smc1Δ/mtrm* females and shown that eggs laid in the last 8 h of the first 24-h brood and the first 8 h of the second 24-h brood are largely responsible for the increased levels of NDJ in aged oocytes (see Fig. S1) and these correspond to oocytes that were in a diplotene-like state when they underwent aging (Subramanian and Bickel 2008).

When we induced the expression of extra SOD1 or SOD2 during meiotic prophase, NDJ was significantly lower than for p{EMPTY} oocytes (genotype *F*_2,305_ = 36.66, *P* < 0.0001). Importantly, we observed an interaction between aging and genotype (aging*genotype *F*_2,305_ = 6.48, *P* = 0.0018) indicating that age-induced NDJ was significantly different for the three genotypes. Compared with p{EMPTY} aged oocytes, p{SOD1} and p{SOD2} aged oocytes exhibited a significant decrease in age-dependent NDJ (Fig. 2). Therefore, the presence of extra SOD protein during meiotic prophase can significantly decrease segregation errors that normally arise due to aging; however, age-dependent NDJ is not completely eliminated in p{SOD1} and p{SOD2} oocytes.

Interestingly, the NDJ values for non-aged oocytes are comparable in all three genotypes. Segregation errors in non-aged oocytes arise primarily from missegregation of achiasmate chromosomes in *mtrm* heterozygotes. Therefore, although a modest increase in SOD during meiotic prophase can act in a protective fashion to lower the incidence of age-induced segregation errors, missegregation events that are not dependent on aging do not appear to benefit from extra SOD.

Like p{EMPTY}, age-induced NDJ in p{SOD1} or p{SOD2} differed across the broods. Although the brood effect was not significantly different between the three genotypes (genotype*brood: *F*_4,305_ = 1.08, *P* = 0.36), one irregularity in the observed pattern is that SOD1 expression appears to have a greater effect on age-induced NDJ in the first brood than the second brood. These data raise the possibility that the attenuation of oxidative damage may be more protective for Drosophila oocytes that are developmentally more mature when they undergo aging. In addition, a comparison of brood 1 data for p{SOD1} and p{SOD2} oocytes indicates that the difference in NDJ between aged and non-aged oocytes is considerably smaller in p{SOD1} oocytes than that in p{SOD2} oocytes. One possibility is that oocytes may benefit more from extra ROS-neutralizing activity in the cytosolic and nuclear compartments than from extra SOD in the mitochondria. However, at this time, we cannot rule out that this observation stems from differences in SOD1 and SOD2 overexpression or their relative enzymatic activities. Further experiments will be required to test these hypotheses.

## Discussion

Our ability to use Drosophila oocytes to model the human maternal age effect has allowed us to test the hypothesis that oxidative stress incurred during aging contributes to age-dependent segregation errors. By using a Gal4/UAS induction strategy, we have specifically increased SOD protein levels in the germline during meiotic prophase and assessed how aging affects the fidelity of meiotic chromosome segregation. Our results indicate that a modest increase in SOD protein in Drosophila diplotene oocytes can significantly reduce the incidence of age-dependent segregation errors. However, the age effect is not completely eliminated in p{SOD} oocytes. Although it is possible that a higher level of SOD expression might have a greater impact, it is also likely that oxidative damage is not the only cause of age-induced segregation errors. Still, our data are consistent with the model that the maternal age effect in humans occurs, at least in part, due to oxidative damage that arises during the aging process which begins at birth.

The proteins that are impacted by oxidative damage in aging oocytes are likely to be numerous and affect multiple pathways. In order to delineate the mechanistic details of this phenomenon, further work will be required to biochemically isolate and identify proteins that become oxidatively damaged during the aging process; such experiments are currently in their early stages in our laboratory. However, the sensitivity of *smc1Δ/+* oocytes to age-dependent NDJ (Subramanian and Bickel 2008) and our previous demonstration that induction of oxidative stress results in premature loss of meiotic cohesion even in the absence of aging (Perkins et al. 2016) suggest that at least some portion of the SOD-mediated suppression of age-induced errors that we observe involves protection of meiotic cohesion. One possibility is that oxidative damage directly impacts the protein linkages that mediate cohesion. However, it is also possible that the effect of oxidative stress is less direct. Further studies will be necessary to investigate these possibilities.

The data we present here, in combination with our previously reported studies (Perkins et al. 2016), support the hypothesis that the accumulation of oxidative damage as human oocytes age contributes to the maternal age effect. Importantly, in our experimental system, age-induced segregation errors can be significantly decreased if extra SOD is available to prevent oxidative damage. Although genetic manipulation of SOD levels in human oocytes is not a viable option, our findings suggest that nutritional supplementation with antioxidants may offer a productive approach to lower the risk of meiotic segregation errors in older women. To begin to explore this possibility, our ability to model the maternal age effect using Drosophila oocytes will be invaluable in asking whether antioxidant supplementation can reduce premature loss of cohesion and segregation errors in aging oocytes. These experiments are currently underway in our laboratory.

## Electronic supplementary material

ESM 1 (PDF 6.44 mb)
