# Supplementary material for: Increased levels of superoxide dismutase suppress meiotic segregation errors in aging oocytes
Source: Chromosoma. 2019 Apr 29;128(3):215–22. doi: 10.1007/s00412-019-00702-y (PMC6823651; doi:10.1007/s00412-019-00702-y)
Supplement: Supplementary file 1 — (PDF 6.44 mb) [file 412_2019_702_MOESM1_ESM.pdf]

Electronic Supplementary Material

Increased levels of superoxide dismutase suppress meiotic segregation errors in aging oocytes

Adrienne T. Perkins*[1,2], Miranda M. Greig*[1], Amrita A. Sontakke[1], Andrew S. Peloquin[1], Mark A. McPeek[1] and Sharon E. Bickel[1]

*equal contributions

[1]Department of Biological Sciences, Dartmouth College, 78 College Street, Hanover, NH 03755

[2]Present address: Interountain Healthcare Precision Genomics, 600 S. Medical Center Drive, St. George, UT 84770

Corresponding Author:
Sharon E. Bickel,
sharon.e.bickel@dartmouth.edu

Table S1: Primer sequences used for cloning SOD1 and SOD2 into pUASP-attB

Table S2: Fly stocks used in this study

Table S3: Type III Tests of Fixed Effects

Fig. S1: Procedure for aging Drosophila oocytes and measuring age-dependent NDJ in broods of progeny.

Fig. S2: Characterization of driver strength for the recombinant *mtrm P{matα-Gal4}* chromosome used in this study.

Fig. S3: Quantification of SOD1 levels in p{EMPTY} and p{SOD1} ovarioles.

References Cited

Table S1: Primer sequences used for cloning SOD1 and SOD2 into pUASP-attB

Primer	Restriction Enzyme Site	Sequence
SOD1 forward	EagI	GTGCGGCCGCAAAATGGTGGTTAAAGCTGTC
SOD1 reverse	XbaI	GCGCGTCTAGAGCTTAGACCTTGGCAATG
SOD2 forward	EagI	GTACGGCCGTCAAAATGTTCGTGGCCCG
SOD2 reverse	XbaI	GCGCGTCTAGAGCTTAGCAACCGAGCTTC

Red indicates restriction enzyme sites and blue denotes Cavener sequence.

Table S2: Fly stocks used in this study

Genotype	Abbreviation	Bickel Stock #	Notes
*y ; p{UASP-**EMPTY** y^+ w^+}attP40 ; smc1^{ex46} / TM3, Ser*	*p{EMPTY}; smc1Δ/TM3, Ser*	I-550	The p{UASP} insertion was generated for this study. *smc1* excision allele (smc1Δ) was obtained from the Hawley lab (now available as Bloomington Stock 25718).
*y ; p{UASP-**SOD1** y^+ w^+}attP40 ; smc1^{ex46} / TM3, Ser*	*p{SOD1}; smc1Δ/TM3, Ser*	I-523	"
*y ; p{UASP-**SOD2** y^+ w^+}attP40 ; smc1^{ex46} / TM3, Ser*	*p{SOD2}; smc1Δ/TM3, Ser*	I-522	"
y / B^SY ; + ; D / TM3, Sb		I-479	
y w / Y ; + ; mtrm^{KG} P{w^{+mC}=matα-GAL4-VP16}V37/TM3, Sb	*mtrm P{matα-Gal4}*	W-073	*mtrm^{KG} matα-Gal4* recombinant chromosome generated in the Bickel lab using *mtrm^{KG}* *(Bloomington* Stock 14932) and *matα-Gal4-VP16 (*Bloomington Stock 7063)<br>"weak driver" used for this study
y w / B^SY ; + ; mtrm^{KG} P{w^{+mC}=matα-GAL4-VP16}V37/TM3, Sb	*mtrm P{matα-Gal4}*	W-107	B^SY derivative of W-073
C(1)RM, y^2, su(w^a) w^a/ X^Y,v f B		C-200	
y w / B^SY ; + ; mtrm^{KG} P{w^{+mC}=matα-GAL4-VP16}V37/TM3, Sb		W-076	*mtrm^{KG} matα-Gal4* recombinant chromosome generated in the Bickel lab as indicated above and utilized previously as a "strong driver" chromosome Perkins, Das, Panzera and Bickel (2016)
w^{1118} ; + ; P{w^{+mC}=UAS-GFP.nls}8		B-101	used to compare the relative strength of Gal4 drivers (Bloomington Stock 4776)

Table S3: Type III Tests of Fixed Effects

Effect	Num DF	Den DF	F value	P < F
Block	1	305	2.30	0.1303
Aging	1	305	83.28	< .0001
Genotype	2	305	36.66	< .0001
Aging*Genotype	2	305	6.48	0.0018
Brood	2	305	8.46	0.0003
Aging*Brood	2	305	9.22	0.0001
Genotype*Brood	4	305	1.08	0.3649
Aging*Genotype*Brood	4	305	2.08	0.0827

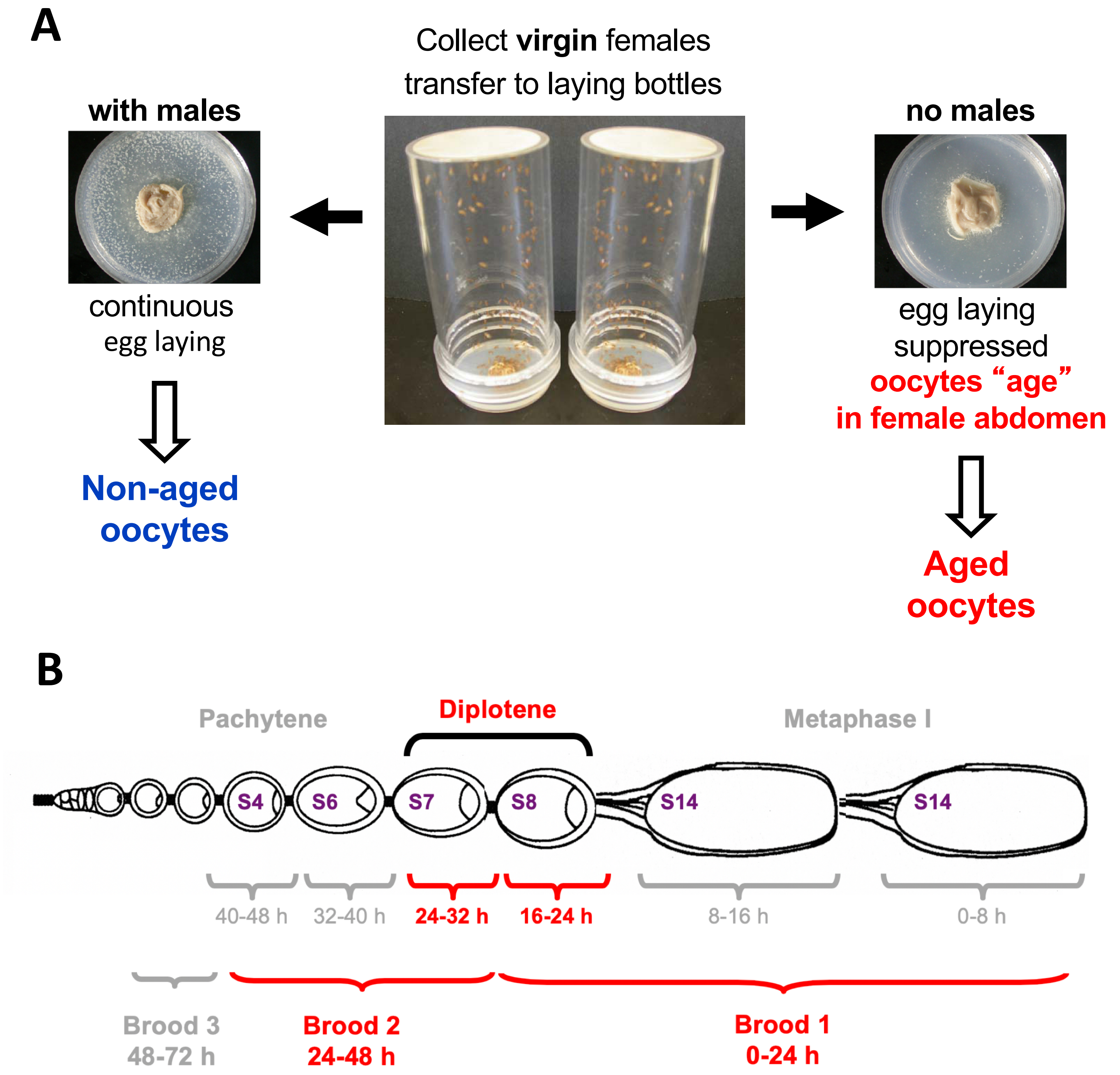

Fig. S1: Procedure for aging Drosophila oocytes and measuring age-dependent NDJ in broods of progeny.
A. For each experiment, virgin females are split into two equal groups. One group is placed into a laying bottle with an equal number of males. These females mate and lay eggs in a continuous fashion, and their oocytes do not undergo aging. The other group is placed into a laying bottle, but no males are added. Egg laying is suppressed in these females and most stages within the ovariole will halt developmental progression and undergo "aging". For both treatments, flies are kept in laying bottles for four days, with fresh plates and yeast paste supplied every 24 hours. At the end of the fourth day, crosses are set up in vials to measure meiotic NDJ in each of the two groups. Upon mating, the females with aged oocytes will commence egg laying. Parents are transferred to new vials every 24 hours to generate "broods" of progeny. The first brood represents the most mature oocytes, which are laid first.
B. For the females subjected to the aging regimen, each brood of progeny corresponds to oocytes that were halted at a specific developmental stage during the aging process. In a previously reported study in which we measured NDJ in 8-hour broods, we demonstrated that the age-dependent NDJ observed in the first two 24-hour broods of *smc1Δ/mtrm* females arises primarily from oocytes that halted and aged at stages 7-8 (Subramanian and Bickel, 2008). These stages correspond to diplotene, the same stage at which human oocytes remain arrested for decades. The ovariole schematic is adapted from Robinson et al. (1994).

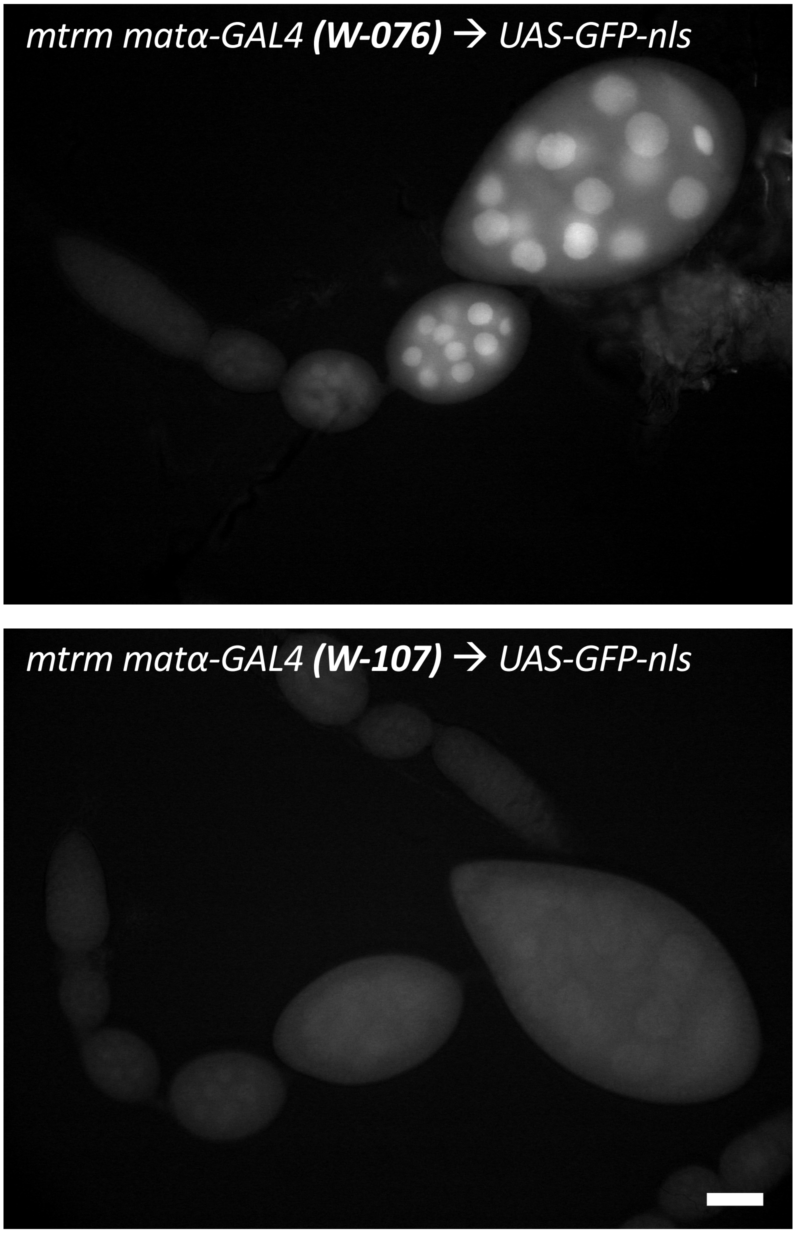

Fig. S2: Characterization of driver strength for the recombinant *mtrm P{matα-Gal4}* chromosome used in this study. Epifluorescence images show the relative Gal4 activity for two different *mtrm P{matα-Gal4}* chromosomes driving expression of a *P{UAS-GFP-nls}* reporter. **Top.** The *mtrm P{matα-Gal4}* recombinant chromosome in the stock W-076 results in robust expression of nuclear-localized GFP signal. Use of this chromosome for RNAi knockdown in the germline has been reported previously (Perkins et al., 2016). **Bottom.** The recombinant *mtrm P{matα-Gal4}* chromosome in stock W-073 (or here, its $B^{S}Y$ derivative W-107) results in much weaker expression of UAS-controlled GFP. For the NDJ tests in this study, the *mtrm P{matα-Gal4}* chromosome in W-073 was used to drive modest overexpression of SOD. Image acquisition and processing were identical for the two images. Scale bar = 30μm.

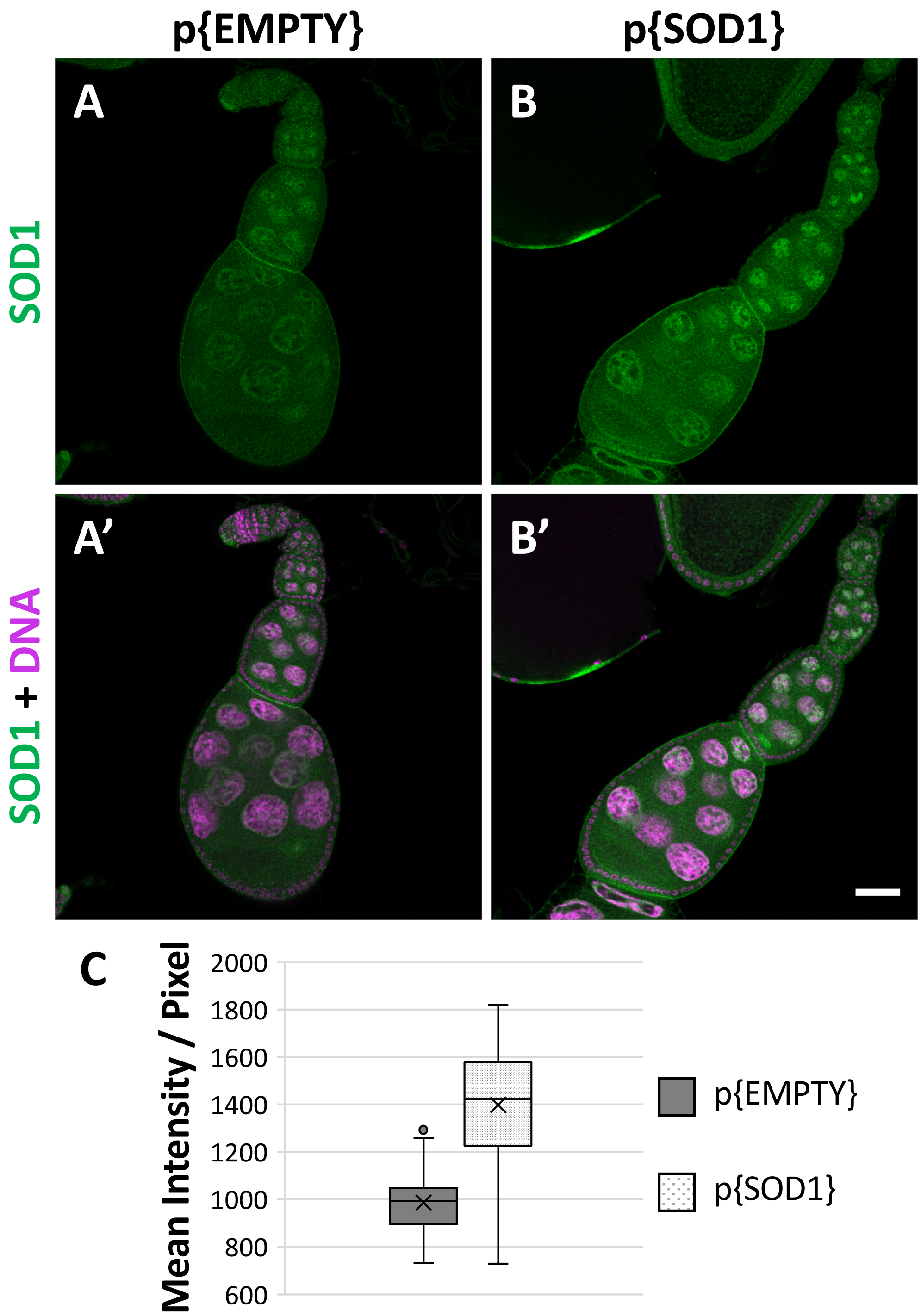

Fig. S3: Quantification of SOD1 levels in p{EMPTY} and p{SOD1} ovarioles. **A & B.** SOD1 immunolocalization (green) is shown for both genotypes. Representative confocal images collected with the same settings are presented. Images are single optical sections. **A' & B'.** DNA signal (magenta) and SOD1 signal are overlaid. Scale bar = 30µm. The SOD1 signal in the p{EMPTY} genotype corresponds to the endogenous SOD1 protein. Note that an increase in the intensity of both cytosolic and nuclear SOD1 is evident in the p{SOD1} ovariole. In this genotype, the matα-Gal4 driver induces expression of extra SOD1. The largest egg chamber in each image corresponds to stage 8. **C.** SOD1 signal intensity was measured for regions of interest (ROIs) that included the germline area for each stage, but not the layer of somatic follicle cells that surround each stage. Quantification of the SOD1 signal for stages 4-8 in multiple ovarioles of each genotype is presented as a Box and Whisker plot. The mean intensity per pixel in each ROI is graphed. Units correspond to the actual intensity values in 12-bit images (range 0-4095). For each plot, the box spans the interquartile range. The line inside the box corresponds to the median and the X inside the box corresponds to the average. The upper and lower quartiles are represented by the lines on either side of the box. The p{EMPTY} data includes an outlier above the top quartile, denoted as a circle. Although the signal intensities vary within each genotype, the average signal intensity for p{SOD1} is significantly higher than that for p{EMPTY} ($P < 0.0001$, unpaired t test). 32 egg chambers were analyzed for p{EMPTY} ovarioles, and 65 egg chambers were quantified for p{SOD1} ovarioles.

References Cited

Perkins AT, Das TM, Panzera LC, Bickel SE (2016) Oxidative stress in oocytes during midprophase induces premature loss of cohesion and chromosome segregation errors. Proc Natl Acad Sci U S A 113: E6823-E6830.

Robinson DN, Cant K, Cooley L (1994) Morphogenesis of Drosophila ovarian ring canals. Development 120: 2015-2025.

Subramanian VV, Bickel SE (2008) Aging predisposes oocytes to meiotic nondisjunction when the cohesin subunit SMC1 is reduced. PLoS Genet 4: e1000263.
